# Surgical wounds healing by Secondary Intention-2 Trial: outcomes and learning from the internal pilot phase and main trial

**DOI:** 10.1186/s40814-025-01658-w

**Published:** 2025-07-02

**Authors:** Catherine Arundel, Sabeen Zahra, Ian Chetter, on behalf of the SWHSI-2 Trial Investigators, Kalpita Baird, Belen Corbacho Martin, Catherine Hewitt, Caroline Fairhurst, Athanasios Gkekas, Andrew Mott, Pedro Saramago Goncalves, Samantha Swan, David Torgerson, Jacqueline Wilkinson, Jane Blazeby, Rhiannon Macefield, Stephen Dixon, Josie Hatfield, Angela Oswald, Matthew Lee, Thomas Pinkney, Nikki Stubbs, Lyn Wilson, Site investigators, A. Clothier, D. Bosanquet, M. Blow, C. Price, J. Todd, T. Munro, W. Pillay, A. Pradhan, A. Garnham, M. Wall, K. Powezka, A. Syed, D. Gerrard, A. Croucher, A. Firth, T. Roe, G. Smith, C. Bicknell, C. Carr, E. Negbenose, L. Tarusan, A. Vesey, D. Wilson, D. Bell, J. Fletcher, C. Greenwood, T. Wallace, S. Vallabhaneni, S. Holder, J. Williams, S. Sim, A. L. Tambyraja, F. Kerray, A. Ng, M. Sylvester, L. Slater, S. T. Rashid, A. Palacios, K. Feld, S. Nandhra, G. Stansby, N. Parr, L. Jones, J. Milne, C. Stubbs, R. Hinchliffe, C. Twine, G. A. Antoniou, C. Corbett, S. Munt, S. Warran, R. Fletcher, W. Al-Jundi, M. Burrows, P. Stather, R. Barnes, T. Woodrow, B. Adams, O. Agu, Y. Gleeson, R. D’Souza, L. Erete, S. Jones, C. Checketts, D. Bajic, R. Matravers, I. Loftus, J. Budge, B. Azhar, R. Hancox, C. Pearce, N. Suggett, A. Whitehouse, G. Kuhan, S. Premnath, N. Dattani, V. Hollings, F. Khasawneh, J. AlShakarchi, E. Packer

**Affiliations:** 1 York Trials Unit, University of York, York, YO10 5DD UK; 2 University of Hull, Hull, HU6 7RX UK; 3 Hull York Medical School, Hull, HU6 7RX UK; 4 Hull University Teaching Hospitals NHS Trust, Hull, HU3 2 JZ UK

**Keywords:** Surgical wounds, Negative pressure wound therapy, Secondary intention, Wound healing, Internal pilot, Pilot projects, Trial design and conduct

## Abstract

**Background:**

Randomised controlled trials are the most rigorous way of investigating the effectiveness of intervention(s) in healthcare settings. During their conduct, trials often run into challenges which if not overcome can lead to significant research waste. Inclusion of a pilot phase provides a way to evaluate elements such as recruitment rate, site set-up and participant follow-up and to address any difficulties early in the trial. The number of trials including a pilot phase is increasing; however, findings are rarely shared in detail, meaning relevant information and learning may not benefit the wider research community. We aimed to report the learning from the SWHSI-2 internal pilot phase, to inform internal pilot trial design and conduct and to also share the subsequent learnings from the main trial phase.

**Methods:**

The design and outcomes of the 6-month internal pilot phase were embedded within the surgical wounds healing by secondary intention trial. The internal pilot phase assessed site set-up, participant randomisation, intervention delivery and follow-up rates using a pre-specified grading. Details of the impact of the pilot phase on, and subsequent changes to, the main trial phase are also presented. We highlighted the challenges faced during the study and detail strategies that were included to minimise or mitigate these.

**Results:**

The trial achieved satisfactory site set-up and intervention delivery levels; however, recruitment and follow-up rates were lower than anticipated. Approval was received from the funder to proceed to the main trial. Following the pilot phase, and continually during the main trial phase, processes and documentation were reviewed, revised and evaluated to mitigate challenges observed in relation to site engagement, participant recruitment and outcome data collection.

**Conclusion:**

Inclusion of an internal pilot enabled early identification of recruitment and retention challenges with a comprehensive suite of interventions subsequently introduced to mitigate these. There was a successful main trial. The findings from this pilot phase add to the evidence base on the design and evaluation of internal pilot phases of a RCT. Future studies including an internal pilot phase should be encouraged to report their experiences for the benefit of others.

**Trial registration:**

ISRCTN26277546. Prospectively registered 25 March 2019, https://www.isrctn.com/ISRCTN26277546

## Background

Randomised controlled trials (RCTs) are the most robust methodology for evaluating effectiveness of healthcare treatments and interventions. These are often complex studies, and their success is dependent on several factors including the operationalisation of trial processes, intervention delivery as planned and successful recruitment and retention of sites and participants [1–5]. If RCTs run into challenges that prevent them from coming to fruition, this can lead to significant research waste [6, 7]. Approaches that facilitate the identification, minimisation and mitigation of difficulties early in RCTs can therefore be beneficial for the ultimate success of the main trial. One such approach includes an initial pilot phase.

Various definitions of pilot studies have been reported [8–10]. The most common accepted definition is a smaller version of the larger study with a focus on trial process uncertainties, e.g. recruitment, retention, intervention delivery and over intervention effectiveness [11–13]. Pilot trials can be ‘external’ where outcome data are not included in the main trial analyses or ‘internal’ where a pilot phase is nested in the main trial, and its data contribute to the overall main trial results. Compared with external pilots, internal pilots have the advantage of efficiency, thus preventing further research waste [14].

The National Institute for Health and Care Research (NIHR), one of the main healthcare funders in the UK, often mandates inclusion of an internal pilot phase in commissioned funding briefs for definitive RCTs. Progression criteria (criteria which need to be met in order to proceed to the main trial), agreed during the application stage, often include the average recruitment rate per site per month, number of sites opened and the total number of participants recruited as a minimum [15].

As a result, the number of RCTs including internal pilots is increasing; however, their findings are rarely disseminated [10, 16], despite previous research encouraging the systematic reporting of the design, results and evaluation of internal pilot trials [14]. Some information from a pilot trial will be condition or intervention specific; however, there is also likely valuable information with relevance to RCTs more broadly, relating, for example, to recruitment or retention processes, randomisation or data collection methods which may be applicable to RCTs overall and so would be of benefit to the wider research community [17]. The dissemination of pilot trial results is also important from an ethical perspective; all clinical research, including internal pilots, carry a risk to participants, and hence, as with full trial results, it is important that this information is shared [18].

The National Institute for Health Research (NIHR) funded Surgical Wounds Healing by Secondary Intention-2 Trial (SWHSI-2) [19], and a multicentre two-arm parallel group RCT, including an internal pilot phase, was designed to assess the clinical and cost-effectiveness of wound care dressings (negative pressure wound therapy vs usual care) for the treatment of open surgical wounds healing by secondary intention. Healing of wounds by secondary intention is common [20] and occurs when either closure of a wound after surgery is not possible or a closed wound breaks down and reopens. In such instances, wounds may be left open to heal through formulation of granulation tissue from the bottom up. Standard wound dressings are widely used to protect the wound during healing. Negative pressure wound therapy is an alternative dressing which uses a controlled vacuum, which is thought to promote wound healing [21]. The SWHSI-2 trial was designed to evaluate the clinical and cost-effectiveness of NPWT as a treatment for SWHSI. The study planned to recruit 696 participants with a SWHSI from UK hospitals and community NHS Trusts, across a 24-month recruitment period, and randomise them to either NPWT or usual care. Participants were followed up for 12 months from randomisation. The trial results will be reported separately.

Given the recommendation for the reporting of internal pilot phases, the design and outcomes of the SWHSI-2 pilot phase and subsequent design changes are presented here. Further changes made to the study during the subsequent main trial phase in response to further challenges are also detailed. The aim is to add to the limited evidence base of reporting internal pilot phases and help to support continued and successful conduct of trials across the research community.

### SWHSI-2 internal pilot phase

SWHSI-2 included a 6-month internal pilot (01 May 2019 to 31 October 2019) to assess site set-up and randomisation, intervention delivery and follow-up rates. A pre-specified traffic light grading system during the pilot phase determined whether the trial should stop (red), continue with modification (amber) or continue unchanged (green) at the end of this phase. These criteria were agreed through discussion with the funder (NIHR) and the Trial Management Group (TMG). The pilot criteria were assessed at the end of the internal pilot phase, with final review including additional activity occurring up to 03.01.2020. Performance against the prespecified criteria is summarised in Table 1.

**Table 1:** SWHSI-2 pilot criteria and performance

**Criteria**		Status
Set up at least 10 sites	Green > 80% Amber 60–80% Red < 60%	Green — 120%, 12 sites opened to recruitment
Randomise 100 participants		Amber — 63%, 63 participants recruited
Intervention delivery within 48 h		Green — 96%, 26 participants received intervention within 48 h (of 27 recruited to intervention)
Non-response rate to postal follow-up for secondary outcome data (3 months)	Green ≤ 15% Amber 15–20% Red > 20%	Red — 38%

When reviewing the outcomes at the end of the 6-month pilot phase, several key factors related to the amber and red ratings were identified (randomisation and follow-up, respectively). For recruitment, these factors included delays in the set-up and receipt of approvals to enable opening initial research sites. This occurred for two sites that had not been open for sufficient time to contribute to recruitment, and a further three sites were open but yet to commence recruitment. While recruitment rate per site per month was not a pre-specified pilot criterion, this was also reviewed by the TMG. When the recruitment rate per site was adjusted to include only those sites which had recruited at least one participant (*n* = 7), the average recruitment rate by site per month rose from 1.08 to 1.83.

The red rating for response rate to follow-up resulted from poor response rates to postal questionnaires sent at 3 months following randomisation to collect patient-reported outcomes (wound infection, quality of life, wound-related pain and resource use). It is however important to note that of the 63 participants randomised into the trial by the end of the pilot phase, only a small number of participants (*n* = 21, 33%) had reached and had sufficient time to receive and respond to the 3-month follow-up. Of the 21 participants, 13 had provided a response (62%). The primary outcome for the study (time to wound healing) was not collected using postal questionnaires but was instead collected during weekly telephone follow-up with participants. While primary outcome rate was not a pre-specified pilot criterion, when reviewed by the TMG, it was noted that there was also no missing time to healing data for participants randomised at that time, resulting in a green rating for the primary outcome.

The SWHSI-2 pilot phase data was reviewed by the Trial Steering (TSC) and Data Monitoring Committees (DMC), with no major concerns raised about study progress, and hence, both committees were agreeable to the study progressing to the main trial phase. Following submission of the pilot phase report to the funder (NIHR HTA), approval was obtained for the study to continue progress to the main trial. The main trial period followed directly from the end of the pilot phase. Due to lower than anticipated recruitment, and the impacts of the COVID-19 pandemic, the study was subsequently extended by 20 months and so ran until 13 January 2024 (recruitment until 13 January 2023, last patient visit 13 January 2024).

### End of pilot phase design changes

Following TMG review of the internal pilot phase data, several changes were made to the study to maximise recruitment and retention. The TMG, TSC and DMC were supportive of the changes being made.

#### 1) Additional sites

As a result of the pilot phase findings, an immediate change to the SWHSI-2 study was an increase in the number of planned recruiting sites to maximise recruitment. Twenty sites were originally proposed; however, following the pilot, addition of four further sites was initially proposed. Ultimately, a total of 29 sites (an additional 9 from that originally planned) were set up to address the shortfall in observed recruitment. The additional sites included sites in Scotland, Wales, and ethnically diverse areas of England to facilitate diversification of recruitment locations and heterogeneity of study participants. By 04.07.2022, all sites were opened to recruitment for the study which provided a minimum of 6 months for all sites to recruit to the study.

#### 2) Site engagement

From October 2019 (6 months into recruitment), bi-monthly site investigator meetings were arranged to share best practices, to solve recruitment or retention problems and to share relevant information either relating to the trial or emerging evidence in relation to the study intervention (NPWT) or open surgical wounds. The meetings also facilitated a routine platform on which to remind sites about the importance of both the study and ensuring treatment equipoise for recruitment.

All members of the research team at each participating site were invited to attend the meetings which were held remotely using video-conference technology. During the study, 23 meetings were held. Primarily attendees were the research or clinical nurses involved in delivering the study locally; however, some principal investigators (surgeons) also attended.

#### 3) Participant engagement

Despite the difficulties with trial recruitment and retention [1–5], evidence to support trial processes remains limited [22]. In February 2020 (10 months into recruitment), two Studies Within A Trial (SWATs) were included in SWHSI-2 to evaluate methods to improve recruitment and retention: an infographic presentation of the participant information sheet vs no infographic at recruitment (SWAT 1) and a postal thank you card or no thank you card during follow-up (SWAT 2).

#### 4) Wound assessments

A face-to-face assessment of surgical site infection was planned at 3 months; however, within a month of study recruitment commencing, difficulties were noted in facilitating participant attendance for this visit. This assessment was therefore removed from the study, but a retrospective infection assessment was retained as part of the post healing assessment visit.

Due to difficulty experienced in facilitating three face-to-face visits following healing, the number of face-to-face visits (including wound photography) was reduced to 1. The remaining two healing visits were completed by telephone call to ensure healing remained confirmed.

Study participants were also asked to take and return an image of their healed wound using guidance developed in conjunction with the SWHSI-2 patient advisory group. This approach helped the study to obtain healing photography from study participants who were unable to attend in person for any reason.

### Further design changes made during the SWHSI-2 main trial

Further changes were also made to the study during the subsequent main trial phase in response to further challenges. These are detailed below to provide valuable learning on trial design and conduct and to add to the limited evidence base of strategies for successful conduct of trials across the research community. The TMG, TSC and DMC were supportive of the changes being made.

#### Study design and process changes during the COVID-19 pandemic

SWHSI-2 recruitment was paused for 5 months (March to July 2020), in agreement with the sponsor and the funder, due to the COVID-19 pandemic. Prior to recommencing recruitment, key study components were assessed with each site: availability of staff to support the study, elective surgeries recommenced, study treatments available, anticipated restart date and local COVID-19 policies. Reconfirmation of local R&D capacity and capability approval was required before the site recommenced recruitment.

To support study delivery, while adhering to ongoing COVID-19 restrictions and impacts, the following four further changes were made to the study to both support recommencement of activity and to minimise the impacts of the pause on the overall study timelines:

#### 1. Remote site set up

Initially, prior to the COVID-19 pandemic, face-to-face on-site training with the local study team was used to maximise engagement and to balance equipoise issues. Subsequently, as the study un-paused to recruitment, following lifting of pandemic restrictions, site set-up also recommenced. Previous research has suggested that remote meetings with sites do not adversely affect study set-up and site activities [23], and given this and the ongoing restrictions in place across the UK, the decision was made to move to remote site set-up. Video call sessions, via Zoom or MS Teams, were arranged with staff at sites to deliver the same training as delivered face to face. The study team felt that remote site setup method was an efficient and less costly way of delivering site training, and so this continued for the remainder of the study. Further research is however required to verify the effectiveness of remote versus on site initiation visits.

#### 2. Increasing participant recruitment

Due to COVID-19 pressures, many nonurgent or planned surgical procedures were postponed resulting in a backlog of surgeries. The recommencement of surgeries across the NHS afforded an opportunity to maximise study recruitment as sites aimed to reduce surgery waiting times. Reopening of sites was therefore planned to be timed with routine activities recommencing to enable this opportunity to be maximised.

#### 3. Coordinating centre support

The COVID-19 pandemic impacted on NHS staffing capacity given increased absences due to sickness and redeployment of staff to treat COVID-19 patients and/or to support prioritised COVID-19-related research. The SWHSI-2 coordinating centre team (York Trials Unit) therefore undertook weekly telephone assessments for recruiting sites where necessary to ensure continued data collection during this time. Due to the pause in site set-up and recruitment activity across trials during this time, this afforded the team capacity to undertake these weekly assessments. The team followed local standard operating procedures for the completion of these visits, developed because of the move to remote working due to pandemic restrictions. Any clinical concerns were fed back to the participating site for further action.

#### 4. Participant follow-up strategies

COVID-19 restrictions further precluded hospital attendance by study participants, and so the post-healing process was further amended in October 2020 (18 months into recruitment) to allow sites to undertake healing confirmation using NHS-approved video call technology, taking a screenshot of the healed wound during the call. To our knowledge, however, this approach was not used by any sites within the study.

The low response rate to follow up questionnaires observed in the pilot remained an issue for SWHSI-2 potentially due to participants being followed up for some time following wound healing. Therefore, further retention strategies were also introduced in October 2020 to try and improve response rates:

- A pen was included with each postal follow-up questionnaire, given evidence of increased retention of approximately 2% with this intervention [24].
  A study newsletter was also developed and sent to participants 2 weeks before their 6-month follow-up questionnaire given this has previously been found to increase response rates [25]. This provided an update on study progress and encouraged participants to complete and return questionnaires.
  Response rates to postal follow-up questionnaires increased by 13% (month 3), 18% (month 6) and 8% (month 12). Change in response rate was calculated as the difference in number of completed responses, of those due and anticipated, between study green light and intervention implantation compared with the number of completed responses, of those due and anticipated, following intervention administration until the end of study follow up. Caution must however be taken in the interpretation of these changes to follow up rates given the inclusion of multiple concurrent strategies intended to improve response rates.

- A follow-up call to participants, by the trial coordinating centre (York Trials Unit), who had not responded to their initial or reminder questionnaire mailings was initiated. Where participants were willing, the questionnaire was completed during the telephone call. Implementing this strategy enabled the collection of data from 59%, 39% and 33% of non-responders at the 3-, 6- and 12-month time points, respectively.
- Monthly email reminders to sites were implemented in February 2021 (approximately halfway through recruitment), to encourage sites to use the weekly assessment telephone contact to remind and encourage study participants to complete and return their postal questionnaires.

#### Study design and process changes (not related to the COVID-19 pandemic)

##### 1. Consent process

In accordance with Good Clinical Practice [26], participants were required to provide written informed consent prior to study participation.

Originally, consent was to be taken by a research nurse or clinician; however, some sites reported that health care assistants also took informed consent on other studies, and that the restriction was limiting the ability to approach and consent all eligible and willing patients. The protocol was amended, 2 years after the start of recruitment, to allow consent to be obtained by any suitably qualified and experienced delegated member of research team (as agreed with local R&D and the study sponsor) which facilitated a more efficient recruitment process at some sites.

##### 2. Follow-up questionnaire changes

The low response rates to postal follow-up questionnaires observed in the pilot phase continued to persist as the study progressed. To try and improve this, several changes were introduced.

Following review by the SWHSI-2 patient advisory group, changes were made to the cover letter used to accompany follow-up questionnaires in October 2021 (approximately 2 years into follow-up). The changes aimed to remind participants of the importance of completing the questionnaires and provided guidance on how to complete the questionnaires depending on the participants current wound status (e.g. healed months previously, healed recently, wound amputated).

Anecdotal evidence, based on participants in response to data collection telephone calls, suggested that some participants felt that they did not need to complete the questionnaire(s) following healing of their SWHSI. Therefore, approximately 3 years into the study follow-up period (October 2022), changes were made to the 3-, 6-, and 12-month questionnaires to place the EQ-5D-5L quality-of-life questionnaire at the start of the booklet given this was relevant for all participants to complete irrespective of healing status. Collection of healthcare resources followed the EQ-5D-5L, with revisions made to focus on NHS resources only (e.g. removal of questions regarding private healthcare). Questionnaires relating to wound infection and wound pain came at the end of the 3-month booklet and were removed from the 6- and 12-month booklets as these measures were irrelevant for participants whose wounds had healed before this assessment point. Change in response rate was analysed in the same way as for the pen and newsletter interventions. The implementation of the revised questionnaires appeared to increase response rates by 7%, 16% and 7% at 3, 6 and 12 months, respectively.

A month later, a compliments slip was also introduced to further remind participants of the importance of completing and returning the questionnaires and to offer options for completion by telephone if preferred. There was no evidence that this further improved questionnaire response rates.

To maximise the collection of primary outcome data (time to wound healing), an additional, site-completed, questionnaire was included in the study in April 2020 (1 year into study follow-up) to collect healing information from participants who withdrew from clinical follow-up but consented to access to healthcare records to confirm wound healing. This questionnaire was further modified in June 2022 (18 months from the end of follow-up) to collect healing status information for active participants as well as those withdrawn but consenting to healthcare records access.

Some strategies appear to show an increase or no change in response rates; however, caution must be taken in interpretation given the inclusion of multiple concurrent strategies intended to improve response rates.

##### 3. Site engagement

A range of strategies to promote site engagement with the study were introduced at various points as the study progressed.

- In March 2022 (10 months before the end of recruitment), a letter from the SWHSI-2 chief investigator was also circulated to surgeons at participating sites to reinforce the importance of equipoise, noting how the support of the clinical community was crucial to the study being able to build robust evidence on intervention effectiveness.
  To supplement this, a clinician infographic was also developed for sites to display in relevant locations as a reminder that the study remained ongoing at the site.
  Average monthly recruitment in the 3 months prior to this intervention was 22 participants per month. Average recruitment between March 2022 and May 2022 (3 months from strategy implementation) dropped slightly to 20 participants per month.
  One-on-one remote meetings with all participating sites were undertaken in September 2022 to maximise recruitment activity in the final four months of the study. Recruitment barriers and facilitators, local study support, documentation queries and central support requirements were discussed which enabled the coordinating centre to target appropriate and timely support to sites to facilitate and expedite recruitment. Average monthly recruitment in the 3 months prior to this intervention was 22 participants per month. Average recruitment between September 2022 and November 2022 (3 months from strategy implementation) increased to 24 participants per month.

- A site-specific newsletter was developed and implemented in December 2021 (approximately a year before recruitment ended) for routine circulation to sites to provide an update on recruitment (both overall and at site) and to reinforce the importance of recruiting to the trial. Average monthly recruitment in the 3 months prior to this intervention was 19 participants per month. Average recruitment between December 2021 and February 2022 (3 months from strategy implementation) increased to 22 participants per month.
- In the final 2 months of the study recruitment period, a competitive recruitment competition was introduced across sites. This appeared to improve recruitment at some sites.

## Discussion

The SWHSI-2 trial internal pilot phase was crucial in identifying and minimising recruitment and retention difficulties observed early in the trial. Impacts of the COVID-19 pandemic resulted in prompt identification of efficient methods to facilitate continued study conduct. The range of interventions and changes implemented affords learning for both future trials in similar populations or using similar interventions and for trials overall [17].

Equipoise imbalance was a key recruitment barrier, arising largely due to the intervention (NPWT) being widely used in the NHS despite limited evidence of effectiveness. Critical assessment of site equipoise from the outset was key to minimising or mitigating this. Studies evaluating widely used interventions with limited effectiveness evidence may therefore benefit from adopting a similar approach.

Continuous peer-to-peer support was crucial in minimising equipoise imbalances throughout the trial with the added benefit of continued study promotion and site engagement. Across the range of interventions used, the central coordinating team worked agilely to adapt to the needs of each participating site. Trialists should therefore consider a range of interventions to maximise engagement, and tailor these, where necessary.

Where subjective outcomes (e.g. wound healing) are used, additional objective outcome assessment (such as photographic assessment) is recommended [27]. Following image quality issues in a previous study [28, 29], a robust procedure was implemented for SWHSI-2. We do not report here the impacts of this procedure on the results of the blinded outcome assessment, given this will be reported separately. Studies using subjective outcomes should consider similar robust processes, and we recommend these are piloted before use.

Participants disengaging with the study following wound healing (primary outcome) resulted in patient-reported outcome response rates being lower than anticipated. Consideration should therefore be given to prioritising key secondary outcomes (e.g. EQ-5D-5L for cost-effectiveness) and providing a range of data collection methods to maximise data collection. The input of patient and public members to these discussions is likely to be helpful.

Finally, internal pilot phase progression criteria should be meaningful for the associated study. The SWHSI-2 pilot phase progression criteria used ‘red (stop)’, ‘amber (review)’ and ‘green (go)’, the details of which were agreed with the funder (NIHR) during the application stage. This approach follows previously recommended guidance [14] and is an approach which has been increasingly used in trials [30].. The SWHSI-2 pilot phase criteria included the follow-up rate for postal questionnaires as a criterion. Given the time to event primary outcome was not postal questionnaire based on reflection, this may not have been the most appropriate criterion; however, its inclusion did afford the opportunity to implement strategies to improve secondary outcome data collection.

### Strengths/limitations

As recommended by Avery et al., [14] we report the design and outcomes of the SWHSI-2 internal pilot phase, and further changes were made during the main trial phase. This adds to the current, limited evidence base regarding pilot trial conduct [10, 16]. Previous pilot phase publications often report only the quantitative analysis of the pilot phase. We have however focussed on the strategies used and the justifications for these to provide useful information for trialists and research stakeholders [17].

This was a retrospective review of the study conducted, and so reporting may be limited given that the activities were not recorded in a structured manner at the time of implementation. Routine funder progress reporting, which documented the strategies used, however minimised this limitation. Studies may therefore benefit from maintaining a structured log of changes made to enable continued evaluation of study processes.

A number of the strategies implemented do not have definitive evidence for their effectiveness, e.g. participant newsletters and site incentives [31, 32], and we did not robustly evaluate these strategies. The changes in recruitment or retention rates following intervention implementation however suggest that interventions may have had a positive direction of effect; however, as several interventions were embedded into the study at the same time (e.g. pens and newsletters), it is not possible to determine individual intervention effectiveness, and so caution must be taken in interpretation of the changes in response rates. Additional robust evaluation of strategies, using randomised study within a trial methodology, is therefore recommended.

## Conclusion

Including a comprehensive suite of interventions to improve recruitment and retention and being agile to the changing barriers and facilitators of a study can be critical to the successful delivery of a RCT. The learning here adds to the limited evidence base on the design and evaluation of internal pilot phases of a RCT, and future studies including an internal pilot phase should also be encouraged to report their experiences for the benefit of others.

## Data Availability

Data sharing is not applicable to this article as no datasets were generated or analysed in relation to this manuscript.

## Abbreviations

DMC: Data Monitoring Committee
EQ-5D-5L: EuroQol 5-Domain 5-Level
NHS: National Health Service
NIHR: National Institute for Health Research
NPWT: Negative pressure wound therapy
RCT: Randomised controlled trial
SWAT: Study Within A Trial
SWHSI: Surgical wound healing by secondary intention
TMG: Trial Management Group
TSC: Trial Steering Committee
UK: United Kingdom
